# Role of Moesin in Advanced Glycation End Products-Induced Angiogenesis of Human Umbilical Vein Endothelial Cells

**DOI:** 10.1038/srep22749

**Published:** 2016-03-09

**Authors:** Qian Wang, Aihui Fan, Yongjun Yuan, Lixian Chen, Xiaohua Guo, Xuliang Huang, Qiaobing Huang

**Affiliations:** 1 Department of Pathophysiology, Key Lab for Shock and Microcirculation Research, Southern Medical University, Guangzhou, 510515, P. R. China

## Abstract

Disorders of angiogenesis are related to microangiopathies during the development of diabetic vascular complications, but the effect of advanced glycation end products (AGEs) on angiogenesis and the mechanism has not been completely unveiled. We previous demonstrated that moesin belonging to the ezrin-radixin-moesin (ERM) protein family protein played a critical role in AGE-induced hyper-permeability in human umbilical vein endothelial cells (HUVECs). Here, we investigated the impact of moesin on AGE-induced HUVEC proliferation, migration, and tubulogenesis. Silencing of moesin decreased cell motility and tube formation but not cell proliferation. It also attenuated cellular F-actin reassembly. Further, phosphorylation of threonine at the 558 amino acid residue (Thr 558) in moesin suppressed AGE-induced HUVEC proliferation, migration, and tube formation, while the activating mutation of moesin at Thr 558 enhanced HUVEC angiogenesis. Further, the inhibition of either RhoA activity by adenovirus or ROCK activation with inhibitor Y27632 decreased AGE-induced moesin phosphorylation and subsequently suppressed HUVEC angiogenesis. These results indicate that the Thr 558 phosphorylation in moesin mediates endothelial angiogenesis. AGEs promoted HUVEC angiogenesis by inducing moesin phosphorylation via RhoA/ROCK pathway.

Pathological angiogenesis is one of the most important microangiopathies in the development of diabetic vascular complications^1^,^2^. Angiogenesis entails degradation of basement membrane, increased vascular permeability, activation, proliferation, and migration of endothelial cells, and finally, the formation and maturation of tubules. Most of the mediators and signals involved in regulation of activation, adhesion, and motility of endothelial cells potentially modulate the development of angiogenesis^3^,^4^.

ERM proteins belong to band four-point-one ezrin, radixin, moesin (FERM 4.1) protein super family^5^,^6^,^7^. These three proteins show a high degree of structural and functional homology between different species^8^,^9^,^10^. They serve as cross-linkers between actin filaments and plasma membrane, and mediate cell adhesion, microvilli formation, cell motility, and signal transduction processes^11^,^12^,^13^,^14^. The phosphorylation of threonine residues (T567 in ezrin, T564 in radixin, and T558 in moesin) activates the ERM proteins^15^,^16^,^17^. The altered conformation following threonine phosphorylation exposes the binding site of ERM to other molecules. Actin microfilament is connected to the cell membrane bridged by ERM protein. The linker effects of ERM alter cell morphology, motility, adhesion, mitosis, and cell polarity^10^. Moesin represents the most important protein in endothelial cells^18^,^19^. The functional change of moesin might alter angiogenesis.

Advanced glycation end products (AGEs) are a heterogeneous group of complex compounds that are formed irreversibly in serum and tissues via a series of non-enzymatic chemical reactions. AGEs play an important role in the development of diabetes and vascular complications, where levels of AGEs are correlated with the severity of complications^20^,^21^. Our previous work demonstrated that AGEs bind with receptor RAGE and activate the RhoA/ROCK and p38MAPK signaling pathways, impairs the barrier function of endothelial cells and increases vascular permeability. We have also shown that AGEs induced moesin 558 threonine phosphorylation, leading to reorganization of F-actin, formation of stress fiber, and disruption of cell-cell connection, resulting in endothelial barrier dysfunction and increased vascular permeability^22^,^23^.

There is an “angiogenic paradox” in diabetes because this disease impairs the angiogenic response depending on the organs involved and disease evolution^24^. AGEs have been associated both with pro- and anti-angiogenic processes^25^,^26^,^27^. While increased vascular permeability and subsequent plasma leakage are often early events of angiogenesis, we presumed that AGEs play a role in pathological angiogenesis and the development of diabetic vascular complications. We hypothesized that moesin and its phosphorylation modulate AGE-induced HUVEC angiogenesis.

Our previous studies have demonstrated that moesin is a typical downstream target of RhoA/ROCK signaling pathway^22^,^23^ and is an important component of fiber pseudopodia. This study also investigated the effect of RhoA-ROCK-induced moesin phosphorylation in AGE-mediated angiogenesis.

AGEs lead to vascular barrier dysfunction and increased vascular permeability. The phosphorylation of moesin is an important link in this process. The enhanced vascular permeability and leakage of plasma proteins create an environment conducive for endothelial cell activation and migration, and initiation of angiogenesis. We hypothesized that moesin is involved in regulation of cell migration and proliferation induced by AGEs. This study focused on HUVECs to investigate the role of AGEs and moesin in angiogenesis to elucidate the mechanism of diabetic microangiopathy, for prevention and treatment.

## Results

### Down-regulation of moesin expression attenuates endothelial migration and tube formation

**Figure 1:**
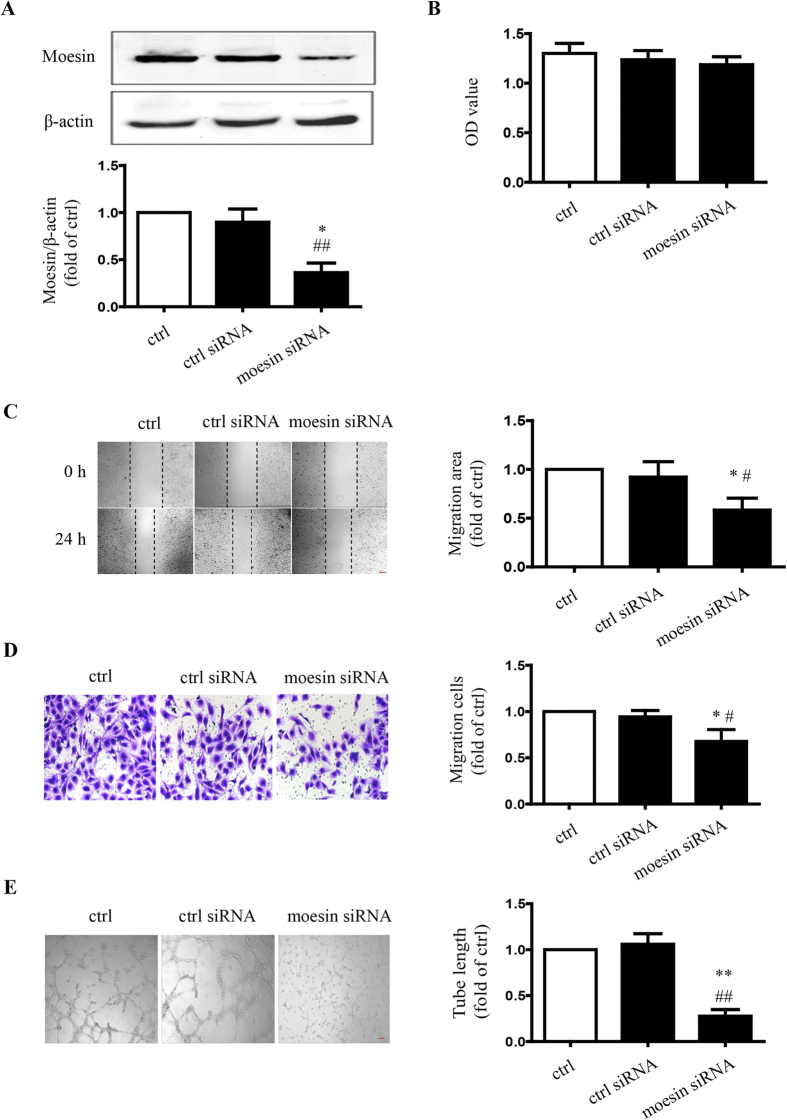
Suppression of moesin expression attenuated endothelial proliferation, migration and tube formation. HUVECs were transfected with control or moesin siRNA for 48 h and the expression of moesin was detected by immunoblotting. The cropped images represent blotting experiments that were performed under the same experimental conditions (**A**). The transfected cells were seeded in plates for measurement of angiogenic parameters. The proliferation of HUVECs was evaluated spectrophotometrically with CCK-8 24 h after seeding (**B**). The migration of HUVECs was measured by scratch wound healing (**C**) and transwell migration (**D**) assays after 24 h of incubation. The tube formation of HUVECs was observed in Matrigel medium after 24 h incubation and the relative lengths of tubes were quantified using Image J (**E**). Scale bar, 100 μm. Data shown are representative of experimental and quantitative results. N = 3 independent experiments. *P < 0.05 vs. control, **P < 0.01 vs. control, ^#^P < 0.05 vs. control siRNA, ^##^P < 0.01 vs. control siRNA.

Initially, the down-regulation of moesin expression by small interfering RNA (siRNA) in HUVECs was confirmed by immunoblotting. Compared with control siRNA, transfection of moesin siRNA for 48 h significantly decreased moesin expression to 36% ± 10% (*P* < 0.05) (Fig. 1A). The effect of moesin suppression on angiogenesis was then evaluated. The viability of HUVECs treated with moesin siRNA was not decreased significantly compared with untransfected or control siRNA treatment (*P* > 0.05) (Fig. 1B). The scratch wound healing and migration assay revealed that compared with untransfected and control siRNA, moesin siRNA transfection remarkably impaired wound healing, as well as attenuated the migration of HUVECs (*P* < 0.05) (Fig. 1C,D). The HUVEC tube formation was also significantly hampered after moesin siRNA treatment (*P* < 0.05) (Fig. 1E). These results indicated that moesin might play an important role in endothelial mobility during angiogenesis.

### Effects of AGEs on endothelial proliferation, migration and tube formation

**Figure 2:**
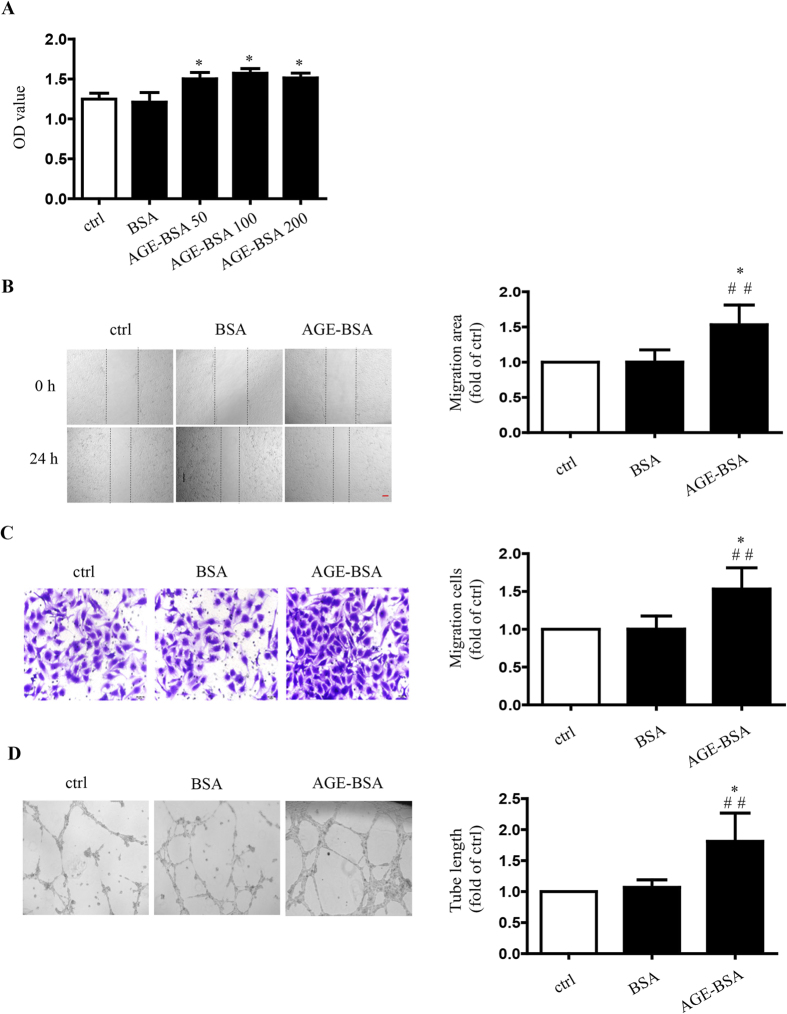
Effects of AGE-BSA on endothelial proliferation, migration and tube formation. The proliferation of HUVECs treated with AGE-BSA in 50, 100 and 200 μg/mL for 24 h was evaluated spectrophotometrically with CCK-8, respectively (**A**). Twenty-four hours after incubation with AGE-BSA (100 μg/mL), the migration of HUVECs was measured by scratch wound healing (**B**) and transwell migration (**C**) assays and, the tube formation was observed in Matrigel medium. The relative lengths of tubes were quantified by using Image J (**D**). Scale bar, 100 μm. Data shown represent experimental and quantitative results. N = 3 independent experiments. *P < 0.05 vs. control, **P < 0.01 vs. control, ^#^P < 0.05 vs. BSA, ^##^P < 0.01 vs. BSA.

To explore the effect of AGEs on endothelial proliferation, migration and tube formation, HUVECs were incubated with 50,100,and 200 μg/mL AGE-modified bovine serum albumin (AGE-BSA) for 24 h. The culture medium was used as a blank control and BSA was used as BSA basal control, respectively. HUVEC proliferation was enhanced after AGE-BSA treatment at all three doses (*P* < 0.05) (Fig. 2A). We selected 100 μg/mL AGE-BSA in subsequent experiments. Compared with blank and BSA control, AGE-BSA accelerated the scratch wound healing process and increased the transwell migration of HUVECs (*P* < 0.05) (Fig. 2B,C). HUVEC tube formation was also enhanced by AGE-BSA (*P* < 0.05) (Fig. 2D). These results indicated that AGEs have the potential to induce angiogenesis.

### Role of moesin in AGE-induced angiogenesis

**Figure 3:**
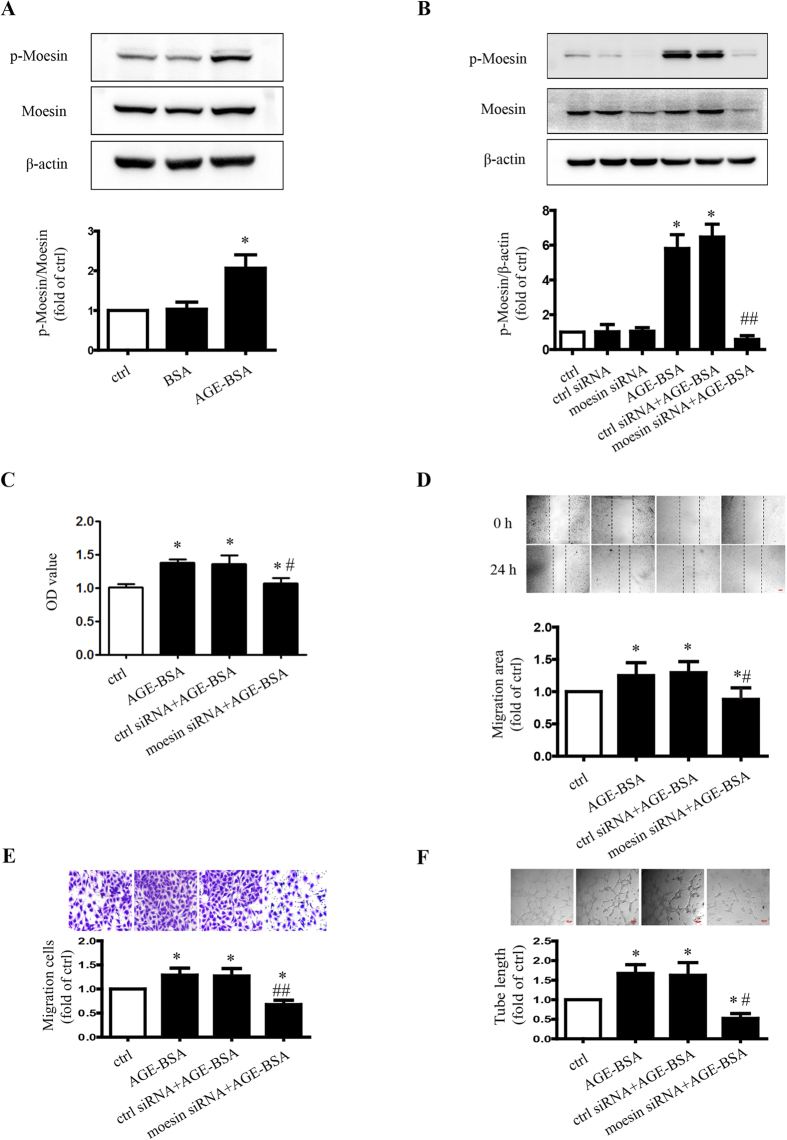
Moesin phosphorylation mediates AGE-induced HUVEC angiogenesis. 24 h after AGE-BSA (100 μg/mL) treatment, moesin phosphorylation in HUVECs was detected using immunoblotting. The cropped images represent blotting experiments that were performed under the same experimental conditions (**A**). HUVECs transfected with control or moesin siRNA for 48 h were treated with AGE-BSA (100 μg/mL) for 24 h. Then moesin phosphorylation (**B**), proliferation (**C**), migration (**D**,**E**) and tube formation (**F**) were measured respectively. Scale bar, 100 μm. Results shown are representative of experimental and quantitative results. N = 3 independent experiments. *P < 0.05 vs. control, ^#^P < 0.05 vs. AGE-BSA, ^##^P < 0.01 vs. AGE-BSA.

Our previous study indicated that AGEs enhance the phosphorylation of moesin. In this study, the results confirmed that AGE-BSA induced moesin T558 phosphorylation, compared with blank control and BSA (*P* < 0.05) (Fig. 3A). Moesin siRNA knocked down the expression of moesin in both BSA and AGE-BSA treated HUVECs. The expression of moesin in BSA with moesin siRNA treatment was down-regulated (Fig. S2A), resulting less basal phosphorylation of moesin, but did not change the ratio of p-moesin/moesin (Fig. S2B). AGE-enhanced moesin phosphorylation was also attenuated significantly as well (Fig. 3B). Down-regulated moesin expression attenuated proliferation (Fig. 3C). It also retarded migration (Fig. 3D,E), and tube formation (Fig. 3F). These results suggested that moesin and its phosphorylation are involved in AGE-induced angiogenesis.

### Role of moesin Thr558 phosphorylation in AGE-induced angiogenesis

**Figure 4:**
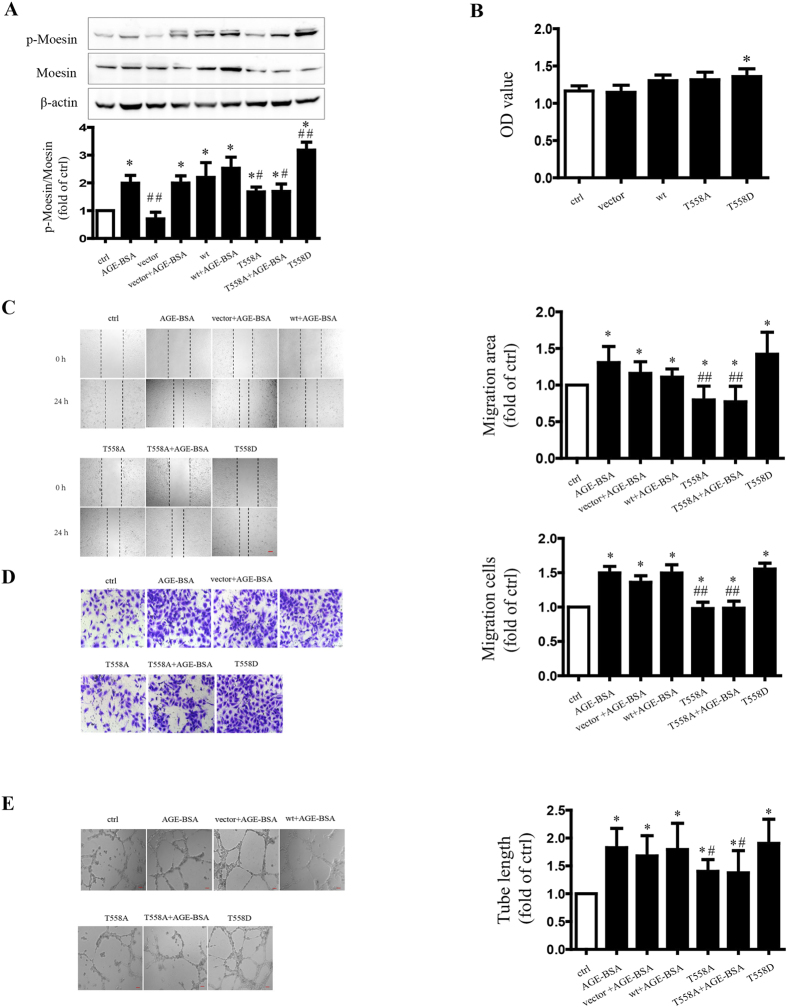
Moesin Thr 558 phosphorylation is important in moesin-mediated angiogenic response. HUVECs were transfected with empty vector, wild type moesin plasmid, pcDNA3/FLAG-moesin^T558A^, respectively, for 24 h with or without AGE-BSA (100 μg/ml,) treatment. pcDNA3.1/FLAG-moesin^T558D^ was transfected into HUVECs without AGE-BSA treatment. Moesin phosphorylation and total moesin expression in HUVECs were detected using immunoblotting. The cropped images represent blotting experiments that were performed under the same experimental conditions (**A**). Twenty-four hour after AGE-BSA treatment, HUVEC proliferation (**B**), migration (**C**,**D**) and tube formation (**E**) were detected respectively. Scale bar, 100 μm. Results shown are representative experiment and quantitative results. N = 3 independent experiments. *P < 0.05 vs control, ^#^P < 0.05 vs AGE-BSA, ^##^P < 0.01 vs AGE-BSA.

We previously reported that moesin phosphorylation of Thr558 residue was important in AGE-induced hyper-permeability in endothelial monolayer^22^. Again, in this study, we confirmed the effect of moesin Thr558 phosphorylation in AGE-induced angiogenesis. The plasmid pcDNA3.1/FLAG-moesin^T558A^ represented inhibited mutant, and pcDNA3.1/FLAG-moesin^T558D^ was the activated mutant, respectively. HUVECs transfected with inhibited mutant pcDNA3.1/FLAG-moesin^T558A^ demonstrated significant attenuation of moesin T558 phosphorylation with or without AGE-BSA application. Transfection HUVECs with activated mutant pcDNA3.1/FLAG-moesin^T558D^ increased moesin T558 phosphorylation automatically. The over-expression of moesin with wild type plasmid mildly increased moesin T558 phosphorylation without AGE-BSA, and resulted in a more significant increase following AGE-BSA addition (Fig. 4A). These results confirmed the effect of AGEs in inducing moesin T558 phosphorylation. Inhibition of mutant moesin did not affect the proliferation of HUVECs, while activating mutant moesin slightly increased the proliferation of HUVECs (Fig. 4B). The migration assay showed that activating mutant moesin T558D enhanced the wound healing and transwell migration of HUVECs. Inhibiting mutant moesin T558A attenuated the wound healing and transwell migration of HUVECs in quiescent stage, as well as during AGE-BSA application (Fig. 4C,D). Similarly, activating mutant moesin T558D enhanced HUVEC tube formation. The inhibiting mutant moesin T558A attenuated the tube formation in HUVECs at the quiescent stage, as well as during AGE-BSA application (Fig. 4E). These results indicate that T558 phosphorylation plays an important role in moesin activation and in AGE-induced angiogenesis.

### Effect of RhoA/ROCK pathway in moesin phosphrylation and AGE-induced angiogenesis

**Figure 5:**
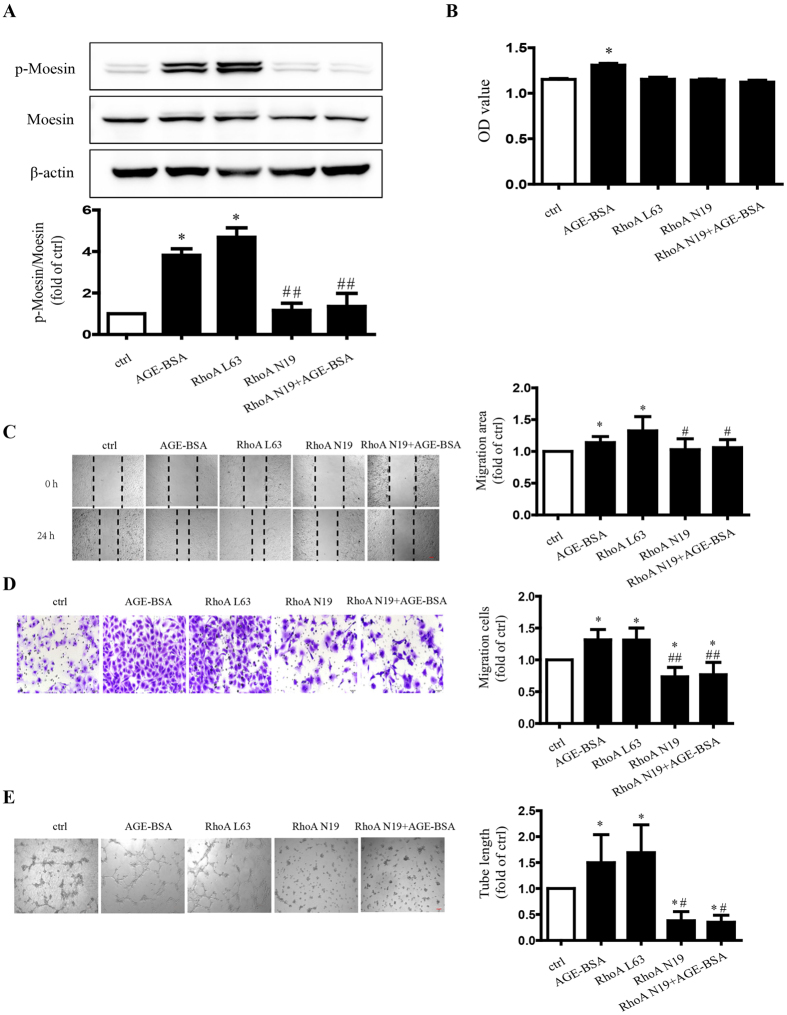
Effects of RhoA on moesin phosphorylation and AGE-induced angiogenesis. Recombinant adenovirus of dominant negative for RhoA (RhoA N19) and constitutively activated RhoA (RhoA L63) were transfected, respectively, into HUVECs for 48 h. AGE-BSA (100 μg/ml) was added in RhoA N19-transfected cells for 24 h. Moesin phosphorylation and total moesin expression in HUVECs were detected using immunoblotting. The cropped images represent blotting experiments that were performed under the same experimental conditions (**A**). The proliferation (**B**), migration (**C**,**D**) and tube formation (**E**) of HUVECs were detected. Scale bar, 100 μm. Results shown are representative experiment and quantitative results. N = 3 independent experiments. *P < 0.05 vs control, ^#^P < 0.05 vs AGE-BSA, ^##^P < 0.01 vs AGE-BSA.

We previously reported that the activation of RhoA/ROCK pathway was critical to moesin phosphorylation and AGE-induced endothelial hyperpermeability^22^,^23^. The data in the present study confirmed that the transfection of dominant negative RhoA (RhoA N19) reduced moesin phosphrylation both in quiescent and in AGE-BSA-stimulated conditions, while constitutively activated RhoA (RhoA L63) transfection enhanced moesin phosphorylation automatically (Fig. 5A). HUVEC proliferation was not affected by manipulation of RhoA activity (Fig. 5B). However, the scratch wound healing and transwell migration were attenuated by down-regulation of RhoA activity with N19 in quiescent and in AGE-BSA-stimulated conditions. The activation of RhoA with L63 demonstrated an enhanced effect similar to AGE-BSA on wound healing and transwell migration (Fig. 5C,D). The tube formation assay revealed relevant results, showing that N19 reduced tube formation in quiescent and AGE-BSA-treated HUVECs and L63 increased HUVEC tube formation (Fig. 5E).

**Figure 6:**
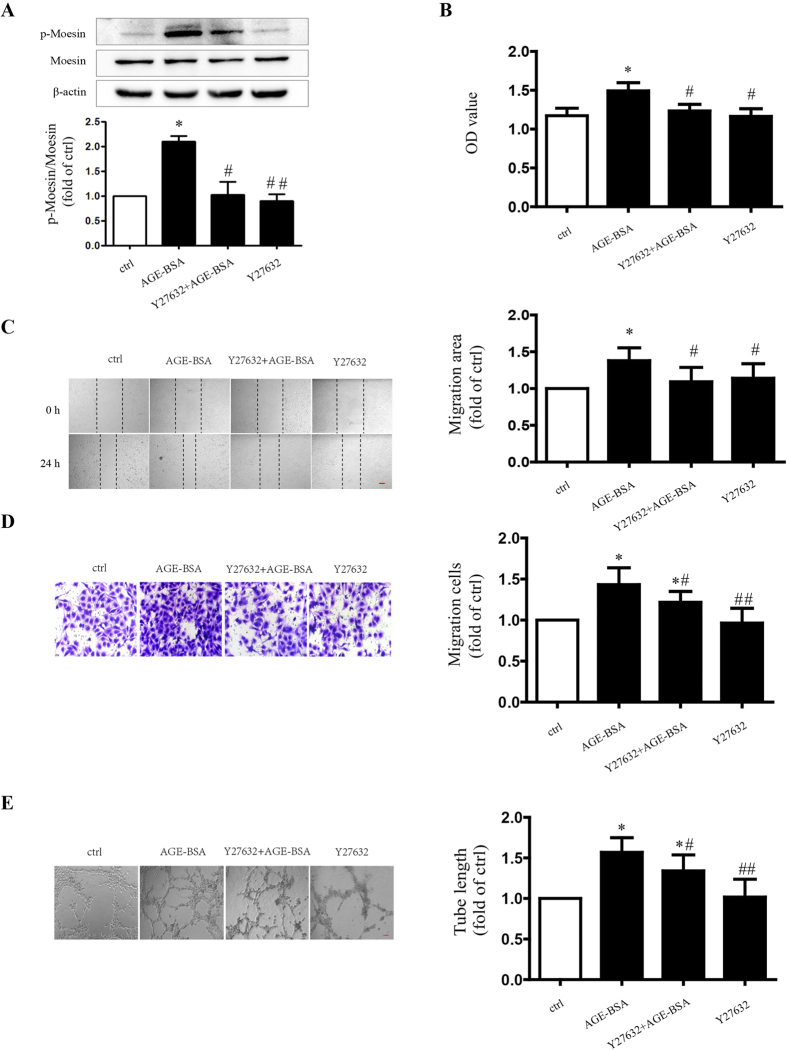
ROCK is involved in moesin phosphorylation and AGE-induced angiogenic responses. Y27632, specific inhibitor of ROCK (10 μmol/L), was administrated 1 h before AGE-BSA (100 μg/ml, 24 h) application. Moesin phosphorylation in HUVECs was detected using immunoblotting. The cropped images represent blotting experiments that were performed under the same experimental conditions (**A**). The proliferation (**B**), migration (**C**,**D**), and tube formation (**E**) of HUVECs were detected. Scale bar, 100 μm. Results shown are representative experiment and quantitative results. N = 3 independent experiments. *P < 0.05 vs control, ^#^P < 0.05 vs AGE-BSA, ^##^P < 0.01 vs AGE-BSA.

The effect of down-stream signaling of RhoA, ROCK, on AGE-induced angiogenesis was also determined in this study. While the inhibition of ROCK with Y27632 significantly attenuated AGE-induced moesin phosphorylation (Fig. 6A), Y27632 also depressed AGE-induced HUVEC proliferation, migration and tube formation as well (Fig. 6B–E). These data demonstrated that RhoA/ROCK activation played a critical role in moesin phosphorylation and AGE-induced angiogenesis.

### Effect of moesin phosphorylation on F-actin assembly

**Figure 7:**
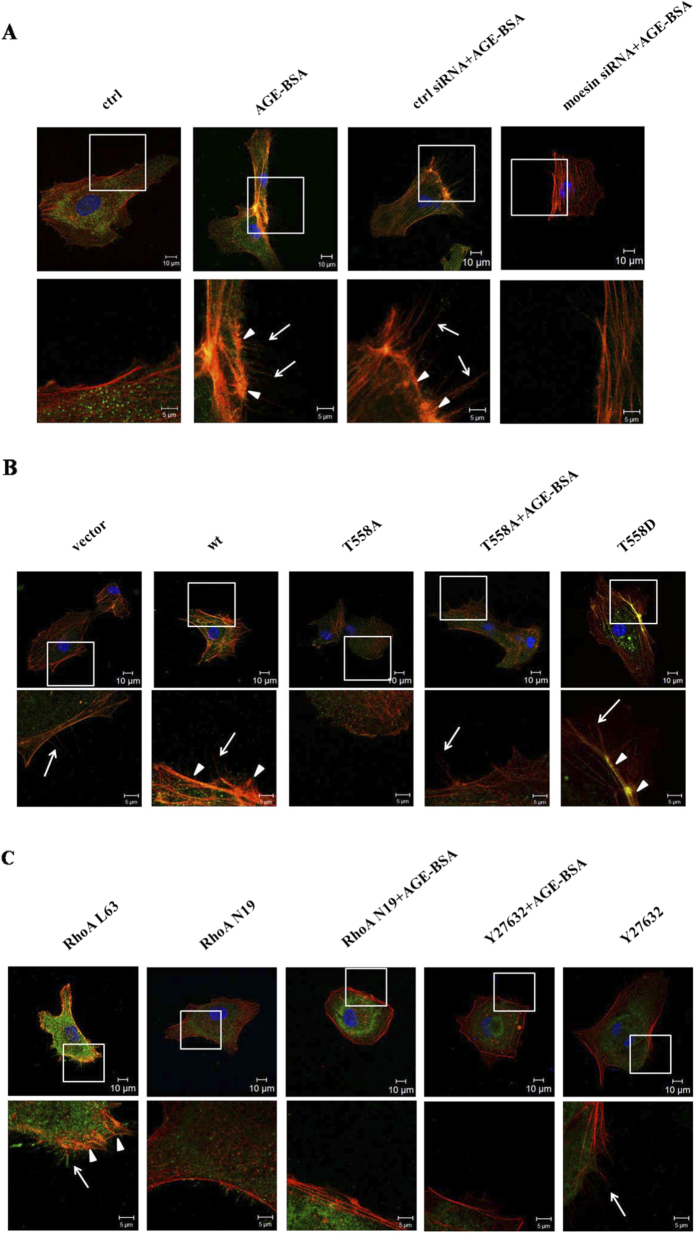
The visualization of p-ERM and F-actin assembly. HVUECs were treated according in different groups. Antibody for p-ERM and FITC-conjugated secondary antibody were used to stain p-ERM (green). Rhodamine-phalloidin and DAPI were used to stain F-actin (red) and nuclear DNA (blue), respectively. The merge images were showed in upper panels and the amplified indicated areas were showed in lower panel in different groups. The filopodia was indicated as sharp spikes (arrow) and lamellipodium (arrow head) was indicated as flat intensive staining. The co-localization of p-ERM and F-actin was visualized by orange color in merge images. Scale bars represent 10 μm. Results shown are representative images in at least 3 experiments.

To further clarify the effect of moesin-altered cell mobility in AGE-evoked angiogenesis, we found altered F-actin distribution and co-localization with phosphorylated-ERM (p-ERM) in the formation of filopodia and lamellipodium. The results showed that compared with control, the immunofluorescent staining of p-ERM was more intense and higher number of filopodia and lamellipodia following AGE-BSA application. The down-regulation of moesin attenuated AGE-induced increase in p-ERM intensity and suppressed the formation of filopodia and lamellipodia (Fig. 7A). The results from transfection of different moesin mutant plasmids further proved that moesin expression was increased with wild type plasmid enhanced phosphorylation of ERM and subsequent formation of F-actin filopodia and lamellipodium. The inhibition of moesin phosphorylation with T558A plasmid decreased AGE-induced formation of filopodia and lamellipodium. In contrast, the activation of moesin phosphorylation with T558D plasmid significantly increased the p-ERM intensity and formation of filopodia and lamellipodium (Fig. 7B). These results morphologically confirmed that moesin phosphorylation at T558 altered AGE-induced cellular mobility. Fluorescent staining after manipulating RhoA and ROCK activities also demonstrated that both RhoA inhibition with adenovirus and ROCK inhibition with inhibitor Y27632 attenuated AGE-induced ERM phosphorylation and formation of filopodia and lamellipodium (Fig. 7C). These results proved, again, that RhoA-ROCK pathway was critical in AGE-induced moesin activation and subsequent cellular morphological changes.

## Discussion

Angiogenesis starts with increased permeability of the basement membrane to enable sprouting of a new capillary, followed by the activation, proliferation, and migration of endothelial cells. Altered cell mobility is one of the initial steps triggering the development of angiogenesis^28^. ERM proteins, especially ezrin and moesin, are involved in cell mobility^29^,^30^. Moesin plays a critical factor in MAP4K4–moesin–talin–β1-integrin molecular pathway to promote efficient plasma membrane retraction during endothelial cell migration, as well as angiogenesis *in vitro* and *in vivo*^31^. This study first aimed to explore the role of moesin in altered endothelial function during the development of diabetic vascular complications. The present study proved that suppression of moesin expression with siRNA attenuated HUVEC migration and tube formation (Fig. 1) without AGE-stimulation, while moesin siRNA had no effect in HUVEC proliferation. These results suggest that moesin plays a critical role in regulating HUVEC angiogenesis by affecting HUVEC motility.

The angiogenic effect of AGEs on ECs has been elucidated for several decades. It has been shown that the accumulation of serum AGEs in diabetes increases the severity of vascular complications^32^. Recent studies have confirmed that the interaction of AGE and receptor RAGE was correlated with angiogenesis^33^,^34^. However, previous reports debated whether AGEs exert pro-angiogenic or anti-angiogenic effects^25^,^27^. Devi and Sudhakaran have demonstrated that different cell environments exert opposite effects on AGE-induced angiogenesis^25^. This study confirmed the pro-angiogenic effects of AGEs in HUVECs by showing that AGE-BSA induced increased angiogenesis (Fig. 3). It is not surprising that AGEs exert pro-angiogenic effects in HUVECs. The increase of hydroimidazolone, one of the components of AGEs, in vitreous humor and serum has been demonstrated to be associated with proliferative diabetic retinopathy (PDR)^35^,^36^.

The mechanisms for AGE-induced angiogenesis have been discussed in various studies^37^,^38^. Our previous studies demonstrated that moesin phosphorylation in endothelial cells plays a key role in AGE-induced endothelial barrier dysfunction and vascular hyper-permeability^22^,^23^. Moesin is a protein linking membrane and cytoskeleton and mediates cytoskeleton-driven cell mobility in angiogenic sprouting^18^,^31^. We, therefore, postulated that moesin may also play an important role in AGE-induced angiogenesis regulation. By down-regulating moesin expression with siRNA and inhibiting moesin phosphorylation with mutant plasmid the present study revealed that inactivation of moesin attenuated AGE-induced HUVEC proliferation, migration and tube formation (Fig. 4). These data further proved that moesin, especially its phosphorylation, mediated AGE-induced angiogenesis. RhoA-ROCK pathway has been reported to be important in regulating endothelial angiogenesis^39^,^40^. We have demonstrated that the interaction between ROCK and moesin and the RhoA-ROCK pathway play a critical role in moesin phosphorylation^23^. The present study provided further evidence supporting the role of RhoA-ROCK in phosphorylated moesin-mediated angiogenesis induced by AGEs (Figs 5 and 6).

Actin and its various protein derivatives are essential for directed cell migration and chemotaxis^41^. Filopodia are thin, actin-rich plasma-membrane protrusions^42^, while lamellipodium, containing a branched dendritic network of actin filaments, is a broad, flat, veil-shaped cell protrusion formed at the leading edge of migrating cells. The formation of stress fibers and cellular contraction is essential for EC migration as well as the angiogeneis process^43^,^44^. Moesin, known as actin-binding protein, is a cytoskeletal protein involved in cytoskeletal changes and paracellular gap formation^45^. Using phalloidin staining of F-actin, the present study visualized increased formation of filopodia and lamellipodium after AGE-BSA application. Using immunofluorescent staining of p-ERM at the same time, this study revealed the co-localization of F-actin and p-ERM and also provided further evidence demonstrating that suppression of moesin expression and phosphorylation attenuated AGE-induced F-actin reassembly and the formation of filopodia and lamellipodium. The inhibition of RhoA-ROCK pathway not only decreased moesin phosphorylation, but also reduced the formation of filopodia and lamellipodium (Fig. 7). These data provided morphological evidence supporting RhoA-ROCK-mediated moesin phosphorylation in AGE-induced angiogenesis. AGEs exert their function by binding with RAGE. Our previous data revealed that RAGE played a critical role in mediating moesin phosphorylation and barrier malfunction in AGE-treated endothelial cells. Recent studies have demonstrated that AGE-RAGE axis also regulates cell migration, invasion, and angiogenesis^33^,^34^. The effect of RAGE in this moesin-mediated angiogenesis in AGE-treated HUVECs needs further investigation.

In conclusion, this study focused on the role of moesin and its phosphorylation in AGE-induced angiogenesis. By providing functional and morphological evidence, the present study demonstrated that Thr 558 phosphorylation of moesin mediates endothelial activation, proliferation, migration, and tubulogenesis. AGEs promoted pathological angiogenesis by inducing moesin phosphorylation through RhoA/ROCK pathway. Moesin is a stably expressed protein in endothelial cells and is located downstream of angiogenic mediating pathways. The complex pro- or anti-angiogenic conditions during the development of diabetic vascular complications suggest that moesin might be a potential therapeutic target in angiogenesis.

## Methods

### Chemicals and reagents

Primary HUVECs and ECM medium were obtained from Sciencell (Carlsbad, CA, USA). Fetal bovine serum (FBS), trypsin, glutamine, penicillin, and streptomycin were all supplied by Gibco BRL (Grand Island, NY, USA). Antibody targeting phosphorylation-moesin (Thr558) was purchased from Abcam (Cat. 2150–1, Cambridge, UK). Antibody recognizing total moesin (Cat.3150), p-ERM (Cat. 3149), total ERM (Cat. 3142), and FLAG were ordered from Cell Signaling Technology (Beverly, MA, USA).

Secondary antibodies for immunoblotting were manufactured by Sigma (St. Louis, MO, USA). FITC-anti-rabbit IgG second antibody was purchased from Molecular Probes, (Cat. A-11008, Life Technologies, Carlsbad, CA, USA) and ROCK inhibitor Y27632 (Cat.Y0503) was acquired from Sigma (St. Louis, MO, USA). The RhoA N19 recombinant adenovirus (dominant negative) and RhoA L63 recombinant adenovirus (constitutively active) were purchased from Cell Biolabs (San Diego, CA, USA). Other chemicals were purchased from Sigma (St. Louis, MO, USA) unless otherwise indicated.

### Preparation of AGE-BSA

AGE-BSA was prepared as previously reported^23^ essentially according to the protocol of Hou *et al.*^46^. Briefly, BSA (150 mmol/L, pH 7.4) was incubated in PBS with D-glucose (250 mmol/L) at 37 °C for 8 weeks, while the control albumin was incubated without glucose. After the incubation period, both solutions were extensively dialyzed against PBS and purified. The endotoxin content was measured with a limulus amoebocyte lysate assay (Sigma, St. Louis, MO, USA) and was found to be less than 500 U/L in both solutions. AGE-specific fluorescence was determined by ratio spectrofluorometry, showing that AGE-BSA contained an AGE content of 74.802 U/mg protein, while native albumin had an AGE content of less than 0.9 U/mg protein.

### Cell culture

Primary HUVECs were maintained in ECM supplemented with 10% FBS, 100 U/mL of penicillin, and 100 μg/mL of streptomycin at 37 °C in a humidified atmosphere with 5% carbon dioxide. In all the experiments, HUVECs harvested between the second and sixth passages were grown to 90% confluence and starved of serum for 12 h before the various treatments.

### HUVEC angiogenesis assay

#### Cell viability assay

The cell viability was measured using CCK-8 (Dojindo Molecular Technologies Inc., Kumamoto, Japan). Cells were seeded in 96-well culture plates and treated accordingly in different groups. The media was removed and CCK-8 (0.5 mg/mL) was added to each well for 4 h. The absorbance was measured at 450 nm. HUVEC proliferation was evaluated directly based on optical density (OD).

#### Endothelial cell migration assay

Scratch wound healing assay was performed by plating a 500 μL HUVEC suspension at 1 × 10^6^/mL in 24-well plates to grow to confluent monolayer. The monolayers were scratched using a 10 μL pipette tip to leave a 500–600 μm gap between the two parts of cell monolayer. The cells were cultured with ECM medium plus respective stimulation in different groups for 24 h. Pictures of wound healing were obtained immediately and 24 h after adding conditioned media. Images were then analyzed using TScratch software^47^ (47). The HUVEC migration area was calculated as: [open image area at 24 h/initial open image area] *100. The initial area of wound scratch in different groups were kept as similar as possible while there was no significant differences between different groups (supplement Fig. S1). HUVEC migration was also assessed using Transwell (Corning, NY, USA) with 8 μm pore-sized filters. First, 100 μL HUVEC suspension at 4 × 10^5^/ml was plated in the upper chamber of the Transwell. To allow cell migration, the ECM medium was added to the lower chamber as a chemoattractant. After 24 h of incubation at 37 °C, the non-migrated cells in the upper chamber were wiped away with cotton swabs and cells that migrated to the lower surface of the filters were fixed and stained with crystal violet, and photographed with a microscope.

#### Capillary tube formation assay

The assay was performed using Matrigel (Corning Inc., Corning, NY, USA) added to a 96-well plate and the gel was allowed to solidify at 37 °C for 1 h. Subsequently, HUVECs were harvested and re-suspended to 2 × 10^5^/ml in ECM medium and 100-μL cell suspension was seeded onto the Matrigel layer. After 24 h of incubation, tubular network structures were visualized and photographed under a phase contrast microscope. The relative lengths of tubes were quantified by Image analysis software (Image J).

### siRNA-mediated knockdown of moesin

Transfection was performed according to the protocols provided by the manufacturer with slight modifications as previous reports^22^,^48^. Briefly, HUVECs were transfected with optimized concentrations of either human moesin siRNA or control nonsense siRNA, purchased from GenePharma Co., Ltd (Shanghai, China). Duplexes of sense 5′-GGGAUGUCAACUGACCUAAdTdT-3′ and antisense 5′-UUAGGUCAGUUG-ACAUCCCdTdG-3′ were used as targeting sequences, which are part of the coding region for *Homo sapiens* moesin 5′-CAGGGATGTCAACTGACCTAA-3′^48^. Forty-eight hours after transfection, whole cell lysates were subjected to immunoblotting with anti-moesin antibody to confirm siRNA-mediated knockdown in moesin expression.

### Immunoblotting assay

Total cellular extracts were prepared by lysis and sonication of the cells in lysis buffer (20 mmol/L Tris pH 7.4, 2.5 mmol/L EDTA, 1% Triton X-100, 1% deoxycholic acid, 0.1% SDS, 100 mmol/L NaCl, 10 mmol/L NaF, 1 mmol/L Na_3_VO_4_) with protease and phosphatase inhibitors. Samples were subjected to SDS-PAGE, and proteins were transferred to polyvinylidene fluoride (PVDF) membranes. Blots were blocked with 5% bovine serum albumin in TBS containing 0.5% Tween 20 (TBS-T) for 1 h and then incubated with an 1:1000 dilution of primary antibody for moesin or p-moesin (Abcam, Cambridge, UK) overnight at 4 °C on a rocker. After three washes for 5 min each with TBS-T, the blots were incubated with a 1:1000 dilution of HRP-conjugated species-specific respective secondary antibody for 1 h at room temperature. After washing three times for 5 min each with TBS-T, protein bands were visualized by chemiluminescence. Densitometric analysis was performed using Kodak IS2000R Imaging Station.

### Site-specific mutagenesis of moesin and transfection of plasmids into HUVECs

The site-specific mutants of moesin were induced according our previous report^23^ with slight modification: pcDNA3/FLAG-moesin^T558A^ as inhibited mutant, or pcDNA3/FLAG-moesin^T558D^ as activated mutant, respectively. The full-length cDNA of moesin was obtained from HUVECs by RT-PCR and was ligated into eukaryotic expression vector pcDNA3/FLAG to obtain the recombinant plasmid pcDNA3/FLAG-moesin. The point mutations of pcDNA3/FLAG-moesin were generated by inverse PCR with a site-specific mutagenesis kit (Toyobo, Japan)^49^. The two mutants, pcDNA3.1/FLAG-moesin^T558A^ and pcDNA3.1/FLAG-moesin^T558D^, were generated using primer T558A forward, 5′GGCCGAGACAAATACAAGGCCCTGCGCCAGATCCG3′, and reverse, 5′CGGATCTGGCGCAGGGCCTTGTATTTGTCTCGGCC 3′, and primer T558D forward, 5′GGCCGAGACAAATACAAGGACCTGCGCCAGATCCGGC 3′, and reverse, 5′CGCCGGATCTGGCGCAGGTCCTTGTATTTGTCTCGGCC 3′, respectively. The mutations were identified by nucleotide sequencing.

The transfection of plasmids was carried out by endofree plasmid midiprep kit. HUVECs cultured to about 90%~95% confluency in 500 μL Opti-MEM medium without antibiotics, were transfected with plasmids encoding FLAG-tagged two mutant forms of moesin (T558A and T558D) using Lipofectamine™ LTX and PLUS™ reagents (Invitrogen, Carlsbad, CA, USA) according to the manufacturer’s instructions. In a 6-well format, 1 μg DNA was incubated with 500 μL Opti-MEM while 8 μL lipofectamine LTX and 2 μL plus reagent were added and left at room temperature for 30 min. The cultured cells were washed once with Opti-MEM. The DNA-lipid complexes were added to the plates and incubated for 48 h, followed by stimulation with AGE-BSA (100 mg/L, 24 h). The cells were then used for immunoblotting and endothelial angiogenesis assays respectively.

### Manipulation of RhoA-ROCK pathway

RhoA activity was downregulated by transfection of recombinant adenovirus that was dominant negative for RhoA (RhoA N19). HUVECs were transfected with adenovirus constitutively activated RhoA (RhoA L63) to up-regulate RhoA activity^23^. Y27632 (10 μmol/L) was used as ROCK-specific inhibitor.

### Immunofluorescent staining of phospho-ERM, F-actin and nuclear DNA staining

HUVECs were layered on gelatin-coated glass-bottomed microwell plates (MatTek, MA, USA) and cultured until confluence. After appropriate treatment, the cells were fixed and permeabilized for 15 min at room temperature in PBS with 3.7% formaldehyde and 0.5% Triton X-100. The cells were washed in PBS twice, blocked in 5% BSA for 1 h, and after a thorough wash with PBS, incubated with p-ERM antibody (1:50) at 4 °C overnight. Due to difficulties with staining of phosphorylated moesin, p-ERM was used instead in this experiment^31^. After a thorough wash in PBS, the cells were stained with an FITC-conjugated secondary antibody (1:200) and conjugated rhodamine-phalloidin (1:100) for 1 h at room temperature. Cells were further incubated with diamidino-2-phenylindole (DAPI, 1:1000) for 15 min and then washed with PBS again. Rhodamine-phalloidin and DAPI were used to stain F-actin and nuclear DNA, respectively, to reveal the location of the cytoplasm and microvilli in the HUVECs. The staining results were imaged using a Zeiss LSM780 laser confocal scanning microscope (Zeiss, Germany).

### Statistical analysis

Data were normalized to control values and reported as a percentage of the baseline values (mean ± SD) for at three independent experiments. Results were analyzed by one-way ANOVA followed by post hoc comparison. The level of significance was set at P < 0.05.

## Additional Information

**How to cite this article**: Wang, Q. *et al.* Role of Moesin in Advanced Glycation End Products-Induced Angiogenesis of Human Umbilical Vein Endothelial Cells. *Sci. Rep.* **6**, 22749; doi: 10.1038/srep22749 (2016).

## Supplementary Material

Supplementary Information
