# Supplementary material for: Role of Moesin in Advanced Glycation End Products-Induced Angiogenesis of Human Umbilical Vein Endothelial Cells
Source: Sci Rep. 2016 Mar 9;6:22749. doi: 10.1038/srep22749 (PMC4783699; doi:10.1038/srep22749)
Supplement: Supplementary Information [file srep22749-s1.pdf]

Role of Moesin in Advanced Glycation End Products-Induced Angiogenesis of Human Umbilical Vein Endothelial Cells

Qian Wang, Aihui Fan, Yongjun Yuan, Lixian Chen, Xiaohua Guo, Xuliang Huang, Qiaobing Huang*

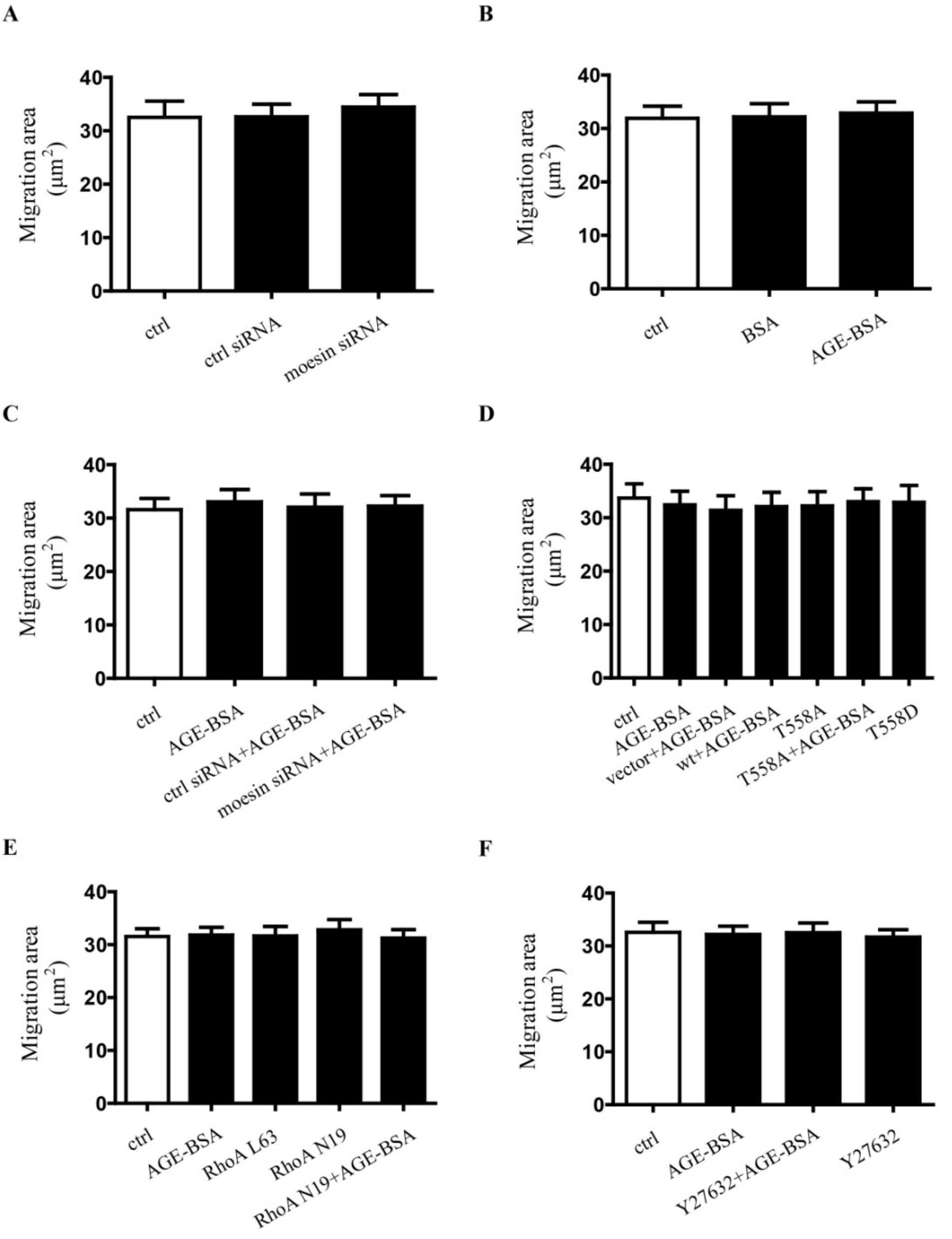

Figure S1. The comparison of initial migration area of HUVECs by scratch wound healing in different treatments. HUVECs were transfected with control or moesin siRNA (A), treated with AGE-BSA (100 μg/mL) (B), transfected with control or moesin siRNA 48 h before AGE-BSA (100 μg/mL, 24 h) treatment (C), transfected with empty vector, wild type moesin plasmid, pcDNA3.1/FLAG-moesinT558A and pcDNA3.1/FLAG-moesinT558D respectively (D), transfected with recombinant adenovirus of dominant negative for RhoA (RhoA N19) and constitutively activated RhoA (RhoA L63) respectively (E) and, treated with ROCK inhibitor Y27632 1 h before AGE-BSA (100μg/mL, 24 h) administration (F). Scale bar, 100 μm. Results shown are representative experiment and quantitative results. N=3 independent experiments.

A

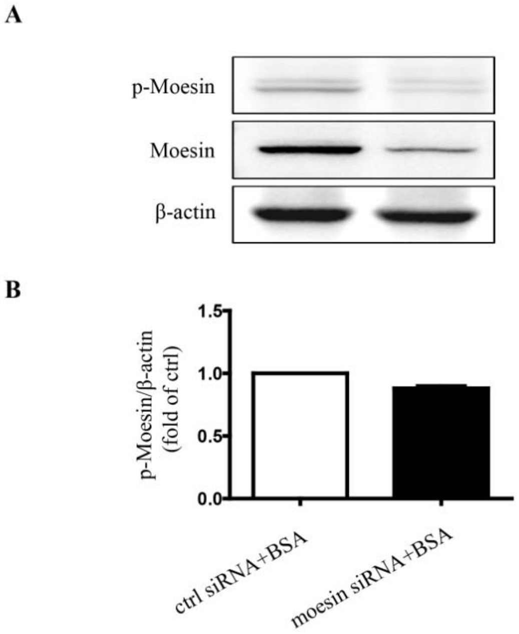

Figure S2. Results of BSA control in moesin expression manipulation. HUVECs transfected with control siRNA or moesin siRNA for 48 h were treated with BSA (100 μg/mL) for another 24 h. The moesin expression and phosphorylation in HUVECs were detected using immunoblotting. The cropped images represent blotting experiments that were performed under the same experimental conditions (A). p-moesin/moesin ratio was also analysed (B). Results shown are representative experiment and quantitative results. n=3 independent experiments.
